# Impact of Lymph Node Burden on Survival of High-risk Prostate Cancer Patients Following Radical Prostatectomy and Pelvic Lymph Node Dissection

**DOI:** 10.3389/fsurg.2016.00065

**Published:** 2016-12-16

**Authors:** Lisa Moris, Thomas Van den Broeck, Lorenzo Tosco, Anthony Van Baelen, Paolo Gontero, Robert Jeffrey Karnes, Wouter Everaerts, Maarten Albersen, Patrick J. Bastian, Piotr Chlosta, Frank Claessens, Felix K. Chun, Markus Graefen, Christian Gratzke, Burkhard Kneitz, Giansilvio Marchioro, Rafael Sanchez Salas, Bertrand Tombal, Henk Van Der Poel, Jochen Christoph Walz, Gert De Meerleer, Alberto Bossi, Karin Haustermans, Francesco Montorsi, Hendrik Van Poppel, Martin Spahn, Alberto Briganti, Steven Joniau

**Affiliations:** ^1^Department of Development and Regeneration, Urology, University Hospitals Leuven, Leuven, Belgium; ^2^Laboratory of Molecular Endocrinology, KULeuven, Leuven, Belgium; ^3^Nuclear Medicine and Molecular Imaging, KULeuven, Leuven, Belgium; ^4^Department of Urology, Maria Middelares, Ghent, Belgium; ^5^A.O.U. San Giovanni Battista-le Molinette, Department of Urology, University of Turin, Turin, Italy; ^6^Department of Urology, Mayo Clinic, Rochester, MN, USA; ^7^Urologische Klinik Und Poliklinik, Klinikum Der Universität München Campus Großhadern, Ludwig Maximilians Universität, Munich, Germany; ^8^Department of Urology, Jagiellonian University Medical College, Krakow, Poland; ^9^Laboratory of Molecular Endocrinology, KULeuven, Hamburg, Germany; ^10^Department of Urology, University of Hamburg, Hamburg, Germany; ^11^Martini Klinik am UKE GmbH, Hamburg, Germany; ^12^Department of Urology and Pediatric Urology, University Hospital Wurzburg, Wurzburg, Germany; ^13^Department of Urology, University of Piemonte Orientale, Novara, Italy; ^14^Department of Urology, Institut Mutualiste Montsouris and Paris Descartes University, Paris, France; ^15^Department of Urology, Cliniques Universitaires SaintLuc, Brussels, Belgium; ^16^Department of Urology, Netherlands Cancer Institute, Amsterdam, Netherlands; ^17^Department of Urology, Institut Paoli Calmettes Cancer Centre, Marseille, France; ^18^Department of Radiation Oncology, University Hospitals Leuven, Leuven, Belgium; ^19^Department of Radiation Oncology, Gustave Roussy Cancer Institute, Villejuif, France; ^20^San Raffaele Hospital, Department of Urology, University VitaSalute, Milan, Italy; ^21^University Hospital Bern, Inselspital, Department of Urology, Berne, Switzerland

**Keywords:** high-risk prostate cancer, lymph node dissection, positive lymph node, prognosis, surgery

## Abstract

**Aim:**

To determine the impact of the extent of lymph node invasion (LNI) on long-term oncological outcomes after radical prostatectomy (RP).

**Material and methods:**

In this retrospective study, we examined the data of 1,249 high-risk, non-metastatic PCa patients treated with RP and pelvic lymph node dissection (PLND) between 1989 and 2011 at eight different tertiary institutions. We fitted univariate and multivariate Cox models to assess independent predictors of cancer-specific survival (CSS) and overall survival (OS). The number of positive lymph node (LN) was dichotomized according to the most informative cutoff predicting CSS. Kaplan–Meier curves assessed CSS and OS rates. Only patients with at least 10 LNs removed at PLND were included. This cutoff was chosen as a surrogate for a well performed PNLD.

**Results:**

Mean age was 65 years (median: 66, IQR 60–70). Positive surgical margins were present in 53.7% (*n* = 671). Final Gleason score (GS) was 2–6 in 12.7% (*n* = 158), 7 in 52% (*n* = 649), and 8–10 in 35.4% (*n* = 442). The median number of LNs removed during PLND was 15 (IQR 12–17). Of all patients, 1,128 (90.3%) had 0–3 positive LNs, while 126 (9.7%) had ≥4 positive LNs. Patients with 0–3 positive LNs had significantly better CSS outcome at 10-year follow-up compared to patients with ≥4 positive LNs (87 vs. 50%; *p* < 0.0001). Similar results were obtained for OS, with a 72 vs. 37% (*p* < 0.0001) survival at 10 years for patients with 0–3 vs. ≥4 positive LNs, respectively. At multivariate analysis, final GS of 8–10, salvage ADT therapy, and ≥4 (vs. <4) positive LNs were predictors of worse CSS and OS. Pathological stage pT4 was an additional predictor of worse CSS.

**Conclusion:**

Four or more positive LNs, pathological stage pT4, and final GS of 8–10 represent independent predictors for worse CSS in patients with high-risk PCa. Primary tumor biology remains a strong driver of tumor progression and patients having ≥4 positive LNs could be considered an enriched patient group in which novel treatment strategies should be studied.

## Introduction

Lymph node (LN) metastasis in men diagnosed with PCa has been shown to be an adverse prognostic factor for biochemical recurrence and survival (1, 2). To determine LN positivity, pelvic lymph node dissection (PLND) is the best staging method (3). Despite its role in staging, the therapeutic effect of PLND is still under debate with no study showing an effect on oncological outcomes (4, 5). This is possibly due to patient heterogeneity and the known variable presentation and evolution of high-risk PCa (6). Therefore, we hypothesized that the extent of lymph node invasion (LNI) might determine the possible therapeutic effect of PLND. A PLND might have a curative effect in patients with a low positive LN burden, whereas patients with a high positive LN burden might have a too high tumor extend for PLND to have an effect. For that reason, we determined the prognostic value of the extent of LNI at PLND on cancer-specific survival (CSS) and overall survival (OS). To achieve this, we examined a contemporary series of patients with high-risk, non-metastatic PCa treated with radical prostatectomy (RP) and PLND at eight different tertiary institutions. We used the number of tumor bearing LNs at final pathology as a surrogate for total LN tumor burden. We decided to focus on high-risk PCa patients since these patients are at an increased risk of PSA failure, the need for secondary therapy, metastatic progression, and prostate cancer-related death (PCRD). In this series, we aimed to determine the cutoff number of positive LNs at which patients shift to a higher risk of PCRD compared to patients without affected LNs.

## Materials and Methods

### Patient Population

We retrospectively analyzed the institutional RP databases of eight different tertiary institutions (Milan, Leuven, Munich, Amsterdam, Novara, Turin, Würzburg, and Krakow) and included all consecutive patients with a negative bone scan and high-risk, non-metastatic PCa defined as minimum stage cT3a OR minimum PSA value of 20 ng/ml OR minimum biopsy Gleason score (GS) of 8. All patients were primarily treated with RP and PLND between 1989 and 2011. As this is a retrospective series studying the role of PLND, there was no reliable data available on the exact anatomical extent of the PLND. It is clear that by removing more LNs and by extending the PLND template, staging accuracy increases significantly (7). Indeed, this is also suggested by our data when analyzing the overall patient population, showing that with an increasing number of LNs removed, the number of positive LNs also increases (Table 1). Based on these results, we decided to only include patients in whom at least 10 LNs were removed at PLND as a surrogate for a well performed PLND. All patients included in this analysis had complete clinical and pathological data, including age at surgery, PSA, preoperative bone scan, pathological stage defined according to the 2010 AJCC staging system (8), specimen GS, surgical margin status, number of LNs removed as well as number of positive LNs, and type of adjuvant treatment. Data on type of salvage therapy were incomplete, with missing data for 271/1,249 patients. The decision to administer adjuvant or salvage therapy [i.e., androgen deprivation therapy (ADT) or radiation therapy (RT)] followed institutional protocols. ADT was generally intended to be lifelong. However, given the retrospective nature of this study, it is uncertain whether patients discontinued treatment after a certain period of ADT. The primary endpoint of the study was CSS and the secondary endpoint was OS, the latter including all men who had not died at last follow-up. Causes of death were determined by the treating physicians and confirmed by death certificates or autopsies if available.

**Table 1:** **Relationship between number of removed lymph nodes (LNs) and number of positive LNs**.

No. of positive LNs (%)	No. of LNs removed					
	1–4	5–9	10–14	15–19	20 or more	
0	299 (90.3)	797 (80.6)	700 (74)	460 (69.4)	501 (53.8)	
1–2	29 (8.8)	142 (14.4)	167 (17.7)	129 (19.5)	215 (23.1)	
3	3 (0.9)	25 (2.5)	31 (3.3)	28 (4.2)	54 (5.8)	
4 or more	0	25 (2.5)	48 (5.0)	46 (6.9)	161 (17.3)	
Total	331 (8.6%)	989 (25.6%)	946 (24.5%)	663 (17.2%)	931 (24.1%)	3,860

### Statistical Analysis

Kaplan–Meier analyses were performed to determine CSS and OS. To determine the positive LN burden most predictive for a worse CSS, our strategy was based on the method used by Abdollah et al. (9). The most informative cutoff predicting a worse CSS was obtained applying the chi-square test for every cutoff value of positive LNs up to the cutoff of ≥4 positive LNs and choosing the cutoff with the lowest *p*-value. We chose to limit the investigated cutoffs up to ≥4 positive LNs, since the incidence of an increasing number of positive LNs becomes exceedingly rare and therefore clinically less relevant. The number of positive LNs was then dichotomized according to the identified cutoff. This cutoff was further validated by univariate and multivariate Cox analyses to determine the prognostic role of positive LN burden on OS and CSS rates, adjusted for covariates. For all statistical analyses, a *p*-value less than 0.05 was deemed statistically significant.

## Results

### Patient Population

Between 1989 and 2011, 4,763 patients with high-risk PCa underwent a RP and PLND at eight different tertiary referral centers. Of these, the number of removed LNs and number of positive LNs were known in 3,860 patients (Table 1). Of the total cohort, 2,540 patients underwent PLND with at least 10 LNs removed. After excluding patients with missing data, we withheld 1,249 patients in our final cohort. The median number of LNs removed during PLND was 15 (IQR 12–17), which correlates with an 80% staging accuracy (7). Of all patients, 47.7% (*n* = 596) had 10–14 nodes removed and 52.3% (*n* = 653) had 15 or more LN removed. In this high-risk PCa cohort, a high rate of LN metastasis (36.1% of patients) was confirmed. In 798 patients, nodal status was pN0 (63.9%); 330 patients had 1–3 positive LN (26.4%) and 126 patients had ≥ 4 positive LN (9.7%) (Table 2).

**Table 2:** **Patient characteristics**.

	Total cohort (*n* = 1,249, 100%)	0 positive lymph node (LN) (*n* = 798, 63.9%)	1–3 positive LN (*n* = 330, 36.4%)	≥4 positive LN (*n* = 121, 9.7%)
**Age**				
Mean	65	65.1	65.1	64.3
Median (IQR)	66 (60–70)	66 (61–73)	65.4 (60–70)	66 (59.8–70)
**Preoperative PSA (ng/ml)**				
Mean	29.8	22.1	31.2	77
Median (IQR)	18.2 (8.1–33.0)	15.2 (7.1–29)	20.8 (10.1–36.8)	35.7 (15.7–64)
**Biopsy Gleason score (GS), no (%)**				
<7	197 (19.8)	165 (20.7)	21 (6.4)	11 (9.1)
7	276 (27.7)	178 (22.3)	76 (23)	22 (18.2)
>7	524 (52.5)	306 (38.3)	156 (47.3)	62 (51.2)
Unknown	252 (20.2)	149 (18.7)	77 (23.3)	26 (21.5)
**cT (UJCC 2002), no (%)**				
cT1–cT2	752 (62.6)	493 (61.7)	195 (59)	64 (52.9)
T3	435 (36.2)	277 (34.7)	117 (35.5)	41 (33.9)
T4	14 (1.2)	0.9 (1)	3 (1)	4 (3.3)
Unknown	48 (3.8)	21 (2.6)	15 (4.5)	4 (9.9)
**Neoadjuvant ADT, no (%)**				
No	1,092 (87.4)	708 (88.7)	280 (84.9)	104 (86)
Yes	134 (10.7)	79 (9.9)	43 (13)	12 (9.9)
Unknown	23 (1.9)	11 (1.4)	7 (2.1)	5 (4.1)
**Surgical margins, no (%)**				
Negative	578 (46.3)	375 (47%)	146 (44.2)	57 (47.1)
Positive	671 (53.7)	423 (53%)	184 (55.8)	64 (52.9)
**pT (UJCC 2002), no (%)**				
pT2	300 (24)	282 (35.3)	14 (4.2)	4 (3.3)
pT3a	384 (30.7)	298 (37.3)	74 (22.4)	12 (9.9)
pT3b	489 (39.2)	198 (24.7)	215 (65.2)	76 (62.8)
pT4	76 (6.1)	20 (2.5)	27 (8.2)	29 (24)
**Final GS, no (%)**				
<7	158 (12.7)	134 (16.8)	16 (4.8)	8 (6.6)
7	649 (52)	471 (59)	145 (43.9)	33 (27.3)
>7	442 (35.4)	193 (24.2)	169 (51.2)	80 (66.1)
**Number of LN removed, no (%)**				
Median	15	15	15	17
10–14	596 (47.7)	395 (49.5)	160 (48.5)	40 (33.1)
≥15	653 (52.3)	403 (50.5)	170 (51.5)	81 (66.9)
**Pathological nodal status, no (%)**				
pN0	798 (63.9)	798 (100%)	–	–
pN1	451 (36.1)	–	330 (100%)	121 (100%)
**Adjuvant therapy**				
RT	118 (9.44)	90 (11.3)	26 (7.9)	2 (1.7)
ADT	221 (17.7)	92 (11.5)	84 (25.5)	45 (37.2)
RT + ADT	183 (14.6)	67 (8.4)	84 (25.5)	32 (26.4)
**Salvage therapy**				
ADT	90 (8.2)	48 (6)	32 (9.7)	10 (8.3)
RT	67 (5.4)	39 (4.9)	21 (2.6)	7 (5.8)
RT + ADT	41 (3.3)	20 (2.5)	16 (2.0)	5 (4.1)
Unknown	271 (21.7)	170 (21.3)	69 (20.9)	32 (26.4)

*ADT, androgen deprivation therapy; RT, radiotherapy*.

### Baseline Patient Characteristics

Baseline demographic data and clinico-pathological characteristics of the 1,249 patients are shown in Table 2. Mean age was 65 years (median 66, IQR 60–70) and mean PSA was 29.8 ng/ml (median 18.2, IQR 8.1–33.0). Positive surgical margins were present in 53.7% (*n* = 671). Pathological T-stage was pT2 in 24% (*n* = 300), pT3a in 30.7% (*n* = 384), pT3b in 39.2% (*n* = 384), and pT4 in 6.1% (*n* = 76). Final GS was 2–6 in 12.7% (*n* = 158), 7 in 52% (*n* = 649), and 8–10 in 35.4% (*n* = 442). Five hundred twenty-two patients (41.7%) received adjuvant treatment immediately after surgery, of whom 221 patients (17.7%) received ADT, 118 patients (%) received RT, and 183 (14.6%) received both ADT and RT. Salvage therapy was given to 198 patients (15.8%), of whom 90 (8.2%) received ADT, 67 (5.4%) received RT, and 41 (3.3%) received both ADT and RT.

### Survival Analysis

**Figure 1:**
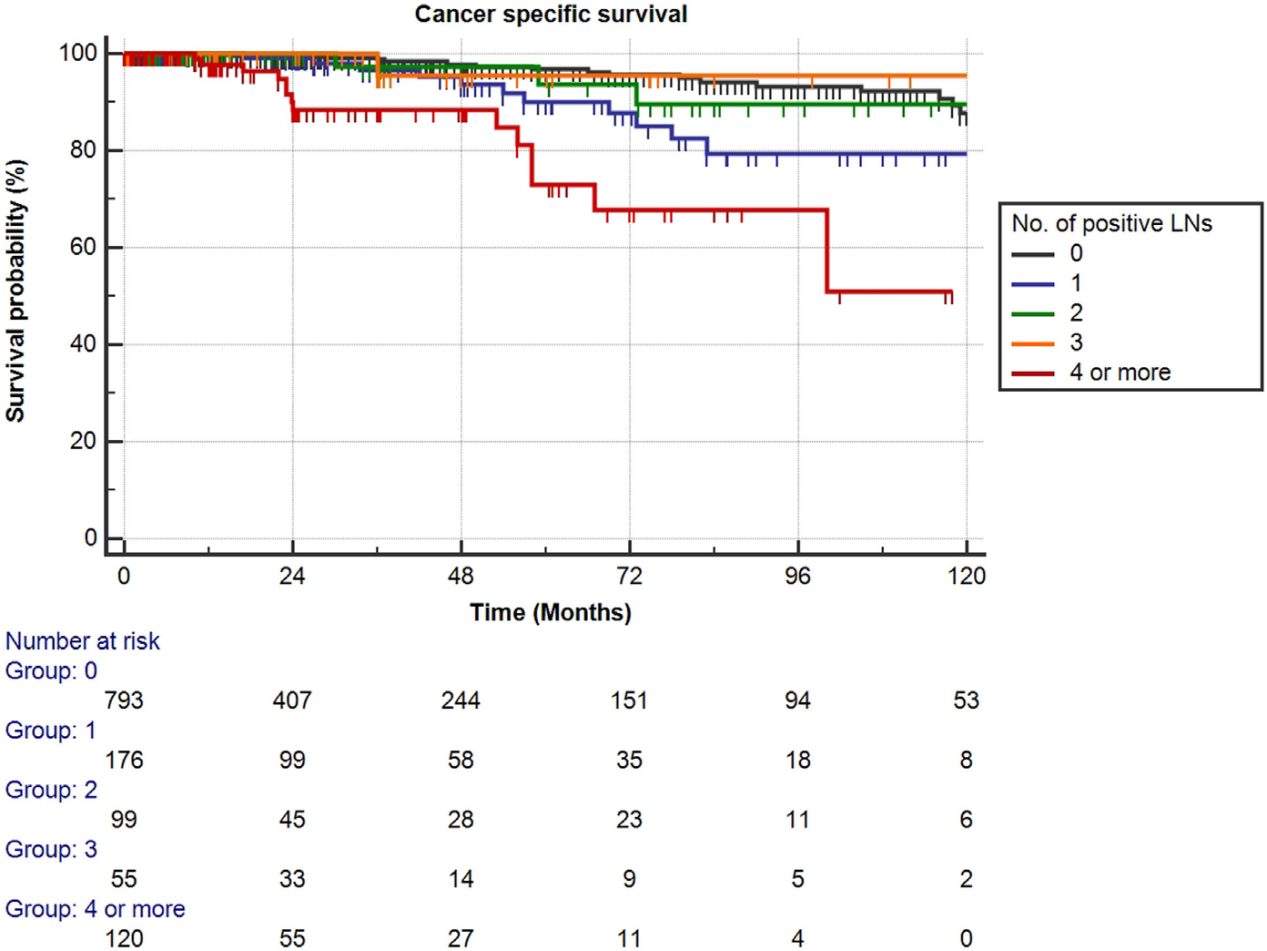
**Kaplan–Meier estimates for cancer-specific survival according to the number of positive lymph nodes (LNs) at pathologic staging**. Black: no positive LN, blue: one positive LN, green: two positive LNs, orange: three positive LNs, and red: four or more positive LNs.

**Figure 2:**
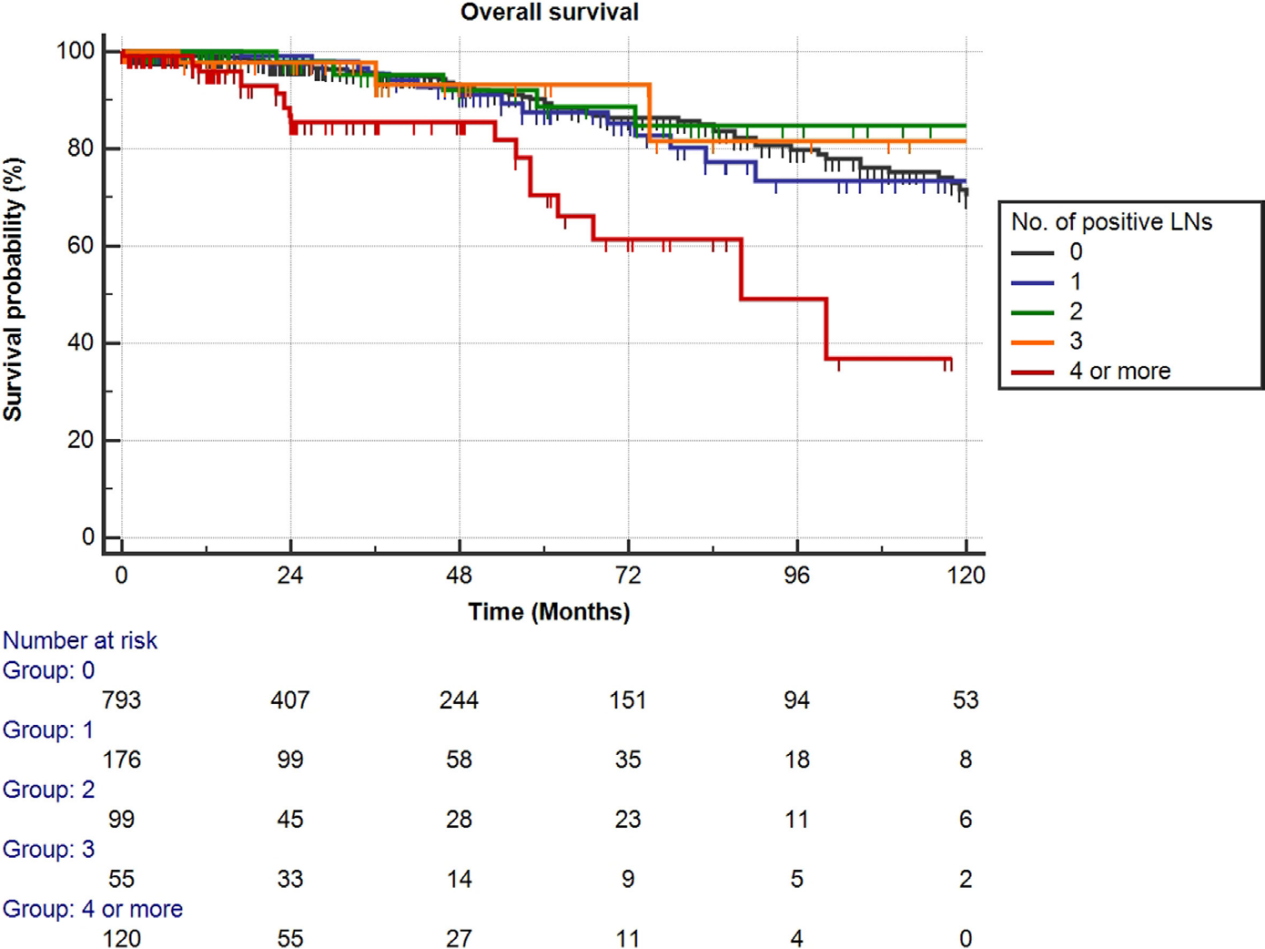
**Kaplan–Meier estimates for overall survival according to the number of positive lymph nodes (LNs) at pathologic staging**. Black: no positive LN, blue: one positive LN, green: two positive LNs, orange: three positive LNs, and red: four or more positive LNs.

**Figure 3:**
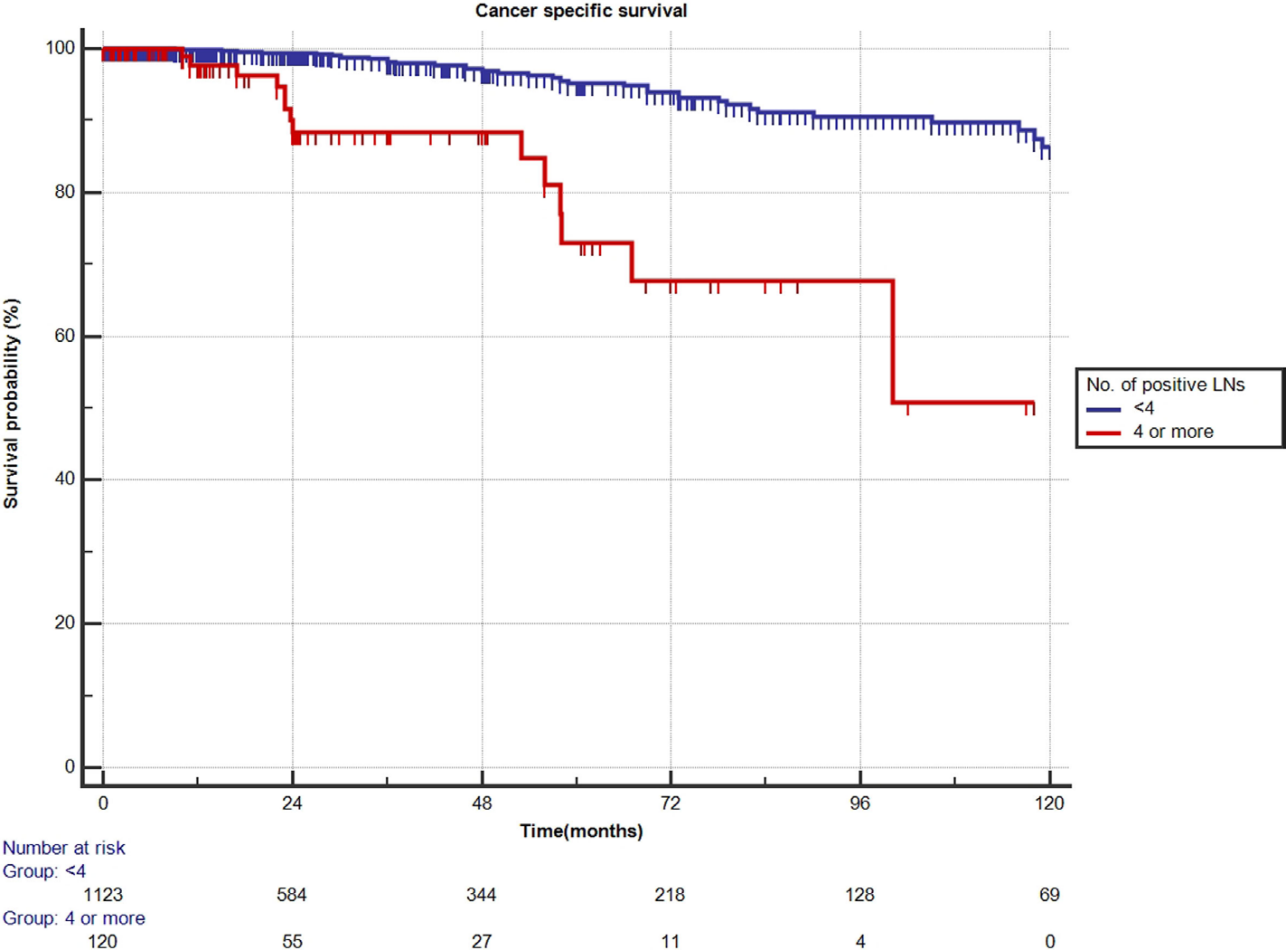
**Kaplan–Meier estimates for cancer-specific survival according to the cutoff of four positive lymph nodes (LNs) at pathologic staging**. Blue: less than four positive LNs and red: four or more positive LNs.

**Figure 4:**
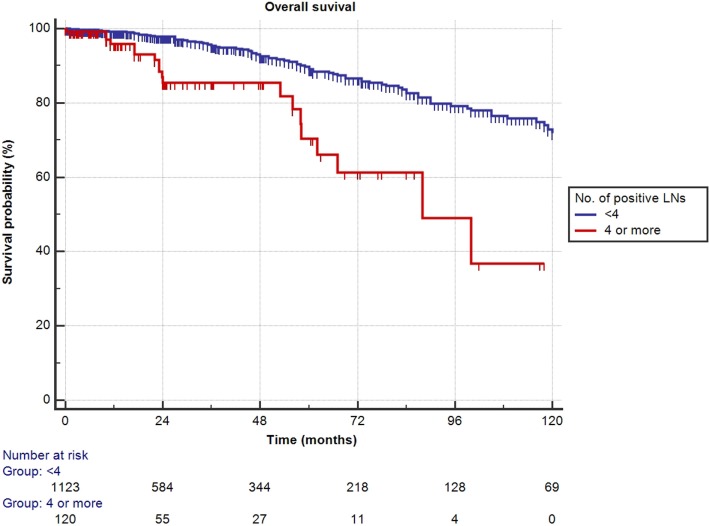
**Kaplan–Meier estimates for cancer-specific survival according to the cutoff of four positive lymph nodes (LNs) at pathologic staging**. Blue: less than four positive LNs and red: four or more positive LNs.

Mean follow-up for survivors was 38.5 months (median 24.3, IQR 11–56). Two hundred eighty-eight patients (23.1%) had a minimum of 5 years follow-up, and 72 patients (5.8%) had a minimum of 10 years follow-up. One hundred nine out of the 1,249 patients had died during data analysis (8.7%), of which 48 patients died from PCa (3.8%). We determined four or more positive LNs as the most informative cutoff of positive LNs predicting PCRD. This was obtained by applying the chi-square test for every cutoff value of positive LNs up to the cutoff of ≥4 positive LNs and choosing the cutoff with the lowest *p*-value (Table 3). Using Kaplan–Meier analysis, estimated 10-year CSS and OS rates are shown in Figures 1 and 2, respectively. We then looked at CSS and OS according to the number of positive LNs using the cutoff of ≥4 positive LNs (vs. <4). The estimated 5-year CSS and OS for 0–3 positive nodes vs. ≥4 positive nodes were 95 vs. 73% and 89 vs. 70%, respectively. Estimated 10-year CSS and OS rates are shown in Figures 3 and 4.

**Table 3:** **Positive lymph nodes (LNs) cutoff to predict cancer-specific survival and overall survival**.

Number of positive LNs	Chi-squared	*p*-Value
≥1 positive LN	11.8	0.0006
≥2 positive LNs	6.4	0.0112
≥3 positive LNs	11.5	0.0007
≥4 positive LNs	20.8	<0.0001

### Cox Regression Analysis for CSS

At univariate analyses, ≥4 positive LNs [hazard ratio (HR): 7.89, *p* < 0.0001], pT3b (HR:13.4, *p* = 0.01), pT4 (HR: 34.22, *p* = 0.0006), pGS 8–10 (HR: 9.39, *p* = 0.0002), positive surgical margins (HR: 3.62, *p* = 0.0001), adjuvant ADT (HR: 2.1, *p* = 0.015), and salvage ADT (HR: 3.27, *p* = 0.004) were predictors of worse CSS (Table 4). At multivariate analysis, pT4 (HR: 2.97, *p* = 0.008), pGS 8–10 (HR: 3.47, *p* = 0.002), salvage ADT (HR: 2.61, *p* = 0.02), and the cutoff of ≥4 positive LNs (HR: 2.74, *p* = 0.03) were confirmed to be independently associated with worse CSS (Table 4).

**Table 4:** **Uni- and multivariate analyses of predictors of a worse CSS**.

	Univariate analysis			Multivariate analysis		
	HR	95% CI	*p*-Value	HR	95% CI	*p*-Value
PSA	1.00	0.99–1.00	NS	–	–	NS
Age	0.99	0.96–1.04	NS	–	–	NS
Ref. pT2	–	–	–	–	–	–
pT3a	4.91	0.61–39.26	NS	–	–	NS
pT3b	13.4	1.81–99.10	0.01	–	–	NS
pT4	34.22	4.53–258.37	0.0006	2.97	1.33–6.62	0.008
Ref. pGS 6	–	–	–	–	–	–
pGS7	2.13	0.59–7.77	NS	–	–	–
pGS 8–10	9.39	2.88–30.62	0.0002	3.74	1.66–8.43	0.002
Positive surgical margins	3.62	1.88–6.9	0.0001	–	–	NS
Ref. <4 positive lymph node (LN)	–	–	–	–	–	–
≥4 positive LN	6.25	3.31–11.79	<0.0001	2.74	1.10–6.83	0.03
Adjuvant androgen deprivation therapy (ADT)	2.1	1.15–3.72	0.015	–	–	NS
Adjuvant radiotherapy (RT)	0.85	0.43–1.66	NS	–	–	NS
Salvage ADT	3.27	1.46–7.35	0.004	2.61	1.15–5.95	0.02
Salvage RT	1.45	0.51–4.15	NS	–	–	NS

*HR, hazard ratio; CI, 95% confidence interval*.

### Cox Regression Analysis for OS

At univariate analyses, ≥4 positive LNs (HR: 3.29, *p* < 0.0001), pT3b (HR: 2.38, *p* = 0.01), pT4 (HR: 4.69, *p* < 0.0001), pGS 8–10 (HR: 2.56, *p* = 0.0004), positive surgical margins (HR: 1.84, *p* = 0.002), and salvage ADT (HR: 2.45, *p* = 0.0015) were predictors of worse OS (Table 5). At multivariate analysis, pGS 8–10 (HR: 2.39, *p* = 0.0003), the cutoff of ≥4 positive LNs (HR: 2.34, *p* = 0.01), and salvage ADT (HR: 2.22, *p* = 0.005) were independently associated with worse OS (Table 5).

**Table 5:** **Uni- and multivariate analyses of predictors of a worse overall survival**.

	Univariate analysis			Multivariate analysis		
	HR	95% CI	*p*-Value	HR	95% CI	*p*-Value
PSA	1.00	0.99–1.01	NS	–	–	NS
Age	1.02	0.99–1.05	NS	–	–	NS
Ref. pT2	–	–	–	–	–	–
pT3a	1.79	0.91–3.55	NS	–	–	NS
pT3b	2.38	1.23–4.60	0.01	–	–	NS
pT4	4.69	2.27–9.72	<0.0001	–	–	NS
Ref. pGS 6	–	–	–	–	–	–
pGS 7	0.99	0.56–1.78	NS	–	–	–
pGS 8–10	2.56	1.51–4.31	0.0004	2.39	1.50–3.82	0.0003
Positive surgical margins	1.84	1.25–2.70	0.0020	–	–	NS
Ref. <4 positive LN	–	–	–	–	–	–
≥4 positive LN	3.41	2.05–5.65	<0.0001	2.34	1.20–4.55	0.01
Adjuvant androgen deprivation therapy (ADT)	1.07	0.73–1.57	NS	–	–	NS
Adjuvant radiotherapy (RT)	0.67	0.41–1.08	NS	–	–	NS
Salvage ADT	2.45	1.41–4.27	0.0015	2.22	1.27–3.88	0.005
Salvage RT	0.84	0.36–1.93	NS	–	–	NS

*HR, hazard ratio; CI, 95% confidence interval*.

## Discussion

Nodal metastases at the time of RP and PLND portend a poor prognosis. However, within the population of node-positive patients, a more detailed risk stratification of survival, based on quantification of positive LN burden, is not incorporated in the 2010 AJCC staging system of PCa. Therefore, the definition of the concept “positive LN burden” remains unclear since controversy remains regarding which characteristics of LN metastasis are actually significant. We focused on high-risk non-metastatic PCa patients, because these patients are at an increased risk of PSA failure, the need for secondary therapy, metastatic progression, and death from PCa. We aimed to compare various risk factors including characteristics of the local tumor, number of positive LNs and therapeutic aspects in a multivariate analysis to determine the prognostic significance of each individual risk factor in predicting CSS and OS. Multiple important observations can be derived from our study.

First, we confirmed overall excellent CSS and OS for patients with high-risk non-metastatic PCa treated with surgery, incorporated within a multimodal treatment approach. In the present cohort, an important role in predicting CSS appears to be represented by the number of positive LNs. Patients with 0 or 1–3 positive LNs had non-different estimated 10-year CSS and OS of 87 and 72%, respectively (Figure 3). Interestingly, only a quarter of the patients with 1–3 positive LNs received adjuvant ADT. About one in every 10 patients (9.7%) had 4 or more positive LNs, and it was only in this subset of patients that 10-year CSS and OS decreased significantly to 50 and 37%, respectively, even though approximately two-third of these patients received adjuvant ADT. A large retrospective analysis from the Mayo Clinic confirmed the finding that RP may offer long-term survival to patients with LN positive PCa, with 10-year CSS as high as 86% (10). In patients identified as LN positive at RP with extended PLND, CSS depended—among other variables—on the degree of LN involvement. The importance of the number of tumor bearing nodes was emphasized again by Briganti et al. Based on their bi-institutional experience including 703 patients, they stated that >2 positive nodes represent a significant cutoff value for CSS in patients with node-positive PCa (11). In both above series, only pN1 patients were included, while patients without LN invasion but having the same primary tumor stage were excluded. By selecting such patient cohort, it is presumed *a priori* that patients with positive LNs have a worse prognosis compared to pN0 patients and thereby rendering a potential bias. Contrary to the above studies, our series did not limit inclusion to pN1 patients. All high-risk PCa patients were equally evaluated, also including those with pN0 disease. This study design allowed us to determine clinical and pathological features predictive for CSS and OS in a real-life clinical situation.

Second, our data show that patients with positive LNs are a highly heterogeneous group. Historically, PCa with positive nodes was considered as a systemic disease for which systemic treatment was required (12). However, our data demonstrate that patients with 0–3 positive nodes had an excellent survival, while those with ≥4 positive nodes had a far worse survival. An individualized and multimodal treatment approach might be more beneficial in this group of patients.

Third, we confirm findings by other groups showing that primary tumor characteristics were highly significant predictors for outcome in addition to positive LNs (10, 13, 14). Indeed, we found advanced pathological stage and pGS 8–10 to be strong predictors of worse CSS and OS. Importantly, pGS 8–10 showed to be an even stronger predictor of worse CSS compared to extensive nodal involvement (≥4 nodes involved). Therefore, we propose that patients with 0–3 positive LNs should be considered having loco-regional disease, with primary tumor characteristics remaining the predominant predictors of survival. Our findings provide a possible explanation for the results of recent studies by Da Pozzo et al., Briganti et al., and Abdollah et al. demonstrating improved survival with adjuvant RT in pN1 patients (15–17). Our observations furthermore add evidence to the concept that in high-risk, non-metastatic PCa, maximal control of the primary tumor may be of higher importance than removal of the LNs. Conversely, in patients with four or more positive LNs, PCa acquires the characteristics of systemic disease. In this subgroup, we hypothesize that the use of a systemic and multimodal treatment might be of greater advantage than salvage radiotherapy alone.

Some strengths of the present study are noteworthy. First, our observations are based upon one of the largest series of high-risk, non-metastatic PCa patients published to date. The results are reinforced by the large number of node-positive patients included and by the fact that these patients were treated in the PSA era, which renders our findings currently applicable. Second, a number of precedent studies used surrogate endpoints, such as biochemical recurrence, to evaluate the prognostic effect of pN1. However, we selected CSS and OS as hard study endpoints in order to attain relevant conclusions. Some studies, which also assessed hard endpoints, only analyzed an LN positive cohort, without taking into account pN0 patients with the same primary tumor stage. Such analyses are biased by presuming upfront that patients with one of the more positive LNs have a worse CSS compared to pN0 (11, 18, 19). To avoid this bias, we included all high-risk PCa patients in our analysis, rather than only pN1 PCa patients and offer the possibility to determine the individual prognostic value of all possible individual covariate risk factors.

However, the results of our study should still be interpreted cautiously. First, our study is limited by biases such as the lack of random assignment, patient selection, incomplete data acquisition, and short time of follow-up. Second, although available for a subset of patients, the exact anatomical PLND template was not captured for the majority of patients. We compensated for this by only selecting patients in whom at least 10 and a median of 15 LNs were removed, which can be considered as a reasonable surrogate for PLND. Nevertheless, due to the wide variation in number of nodes in the primary landing sites, the number of examined nodes might not be the best surrogate for the extent of the PLND and boundaries of the dissection template might be at least as important in defining whether all primary landing sites are removed (19, 20). Finally, data were generated over a long period of time with an unknown number of surgeons, various protocols for pathological assessment of the lymphadenectomy specimens, and varying approaches to the initiation and the continuation of adjuvant treatment. Although we aimed to evaluate the potential survival benefit according to the number of tumor bearing nodes, information of other LN-related factors such as nodal tumor volume, extranodal extension, lymphovascular invasion, and tumor differentiation were not available and hence were not taken into account.

Despite these limitations, this large retrospective analysis is hypothesis generating and should stimulate prospective studies stratifying disease management according to low- vs. high-volume positive LN burden with more aggressive local treatment in low-volume positive LN burden and more aggressive systemic treatment in patients with high-volume positive LN burden.

## Conclusion

Our results show favorable long-term survival outcomes for patients with non-metastatic high-risk prostate cancer. At multivariate analysis, 4 or more positive LNs represented an independent predictor of worse CSS and OS, while patients with 0 vs. 1–3 positive LNs experience similar excellent CSS. Interestingly, advanced pathological stage and final GS of 8–10 were additional important outcome predictors of worse CSS. These observations point to the importance of maximizing local control in high-risk PCa with 0–3 positive LNs. On the contrary, patients having ≥4 positive LNs might be considered as having systemic disease and can be considered as an enriched patient group in which novel treatment strategies should be studied.

Although confirmation by prospective studies is needed, these results suggest that risk stratification and therapy choice should be individualized taking into account their LN status.

## Ethics Statement

We declare that prior to the start of the study, all participating centers have attained ethical committee approval by their institutional review boards. Because of the retrospective nature of the study with long-term follow-up, the need for informed consent was waived by the ethical committees. The study was conducted in accordance with the Declaration of Helsinki (amendment by 64th WMA General Assembly, Fortaleza, Brazil, October 2013).

## Author Contributions

LM, TB, SJ: protocol/project development, data collection or management, data analysis, manuscript writing/editing. LT: data collection or management, manuscript writing/editing. AVB: protocol/project development, data collection or management, manuscript writing/editing. PG, RK, PB, PC, FC, FKC, WE, MA, MG, CG, BK, GM, RS, BT, HvdP, JW, GDM, ABossi, KH, FM, HVP, MS, ABriganti: data collection or management.

## Conflict of Interest Statement

This research received no specific grant from any funding agency in the public, commercial, or not-for-profit sectors. The authors declare no conflicts of interest in preparing this article.
